# Comparison of Hyperspectral Imaging and Microvascular Doppler for Perfusion Monitoring of Free Flaps in an In Vivo Rodent Model

**DOI:** 10.3390/jcm11144134

**Published:** 2022-07-16

**Authors:** Philipp Becker, Sebastian Blatt, Andreas Pabst, Diana Heimes, Bilal Al-Nawas, Peer W. Kämmerer, Daniel G. E. Thiem

**Affiliations:** 1 Department of Oral and Maxillofacial Surgery, Federal Armed Forces Hospital, Rübenacherstr. 170, 56072 Koblenz, Germany; andreas.pabst@uni-mainz.de; 2 Department of Oral and Maxillofacial Surgery, University Medical Centre Mainz, 55131 Mainz, Germany; sebastian.blatt@unimedizin-mainz.de (S.B.); diana.heimes@unimedizin-mainz.de (D.H.); bilal.al-nawas@unimedizin-mainz.de (B.A.-N.); peer.kaemmerer@unimedizin-mainz.de (P.W.K.); daniel.thiem@uni-mainz.de (D.G.E.T.)

**Keywords:** hyperspectral imaging, free flap, perfusion monitoring, MDC, micro-Doppler sonography, rodents

## Abstract

To reduce microvascular free flap failure (MFF), monitoring is crucial for the early detection of malperfusion and allows timely salvage. Therefore, the aim of this study was to evaluate hyperspectral imaging (HSI) in comparison to micro-Doppler sonography (MDS) to monitor MFF perfusion in an in vivo rodent model. Bilateral groin flaps were raised on 20 Sprague–Dawley rats. The femoral artery was transected on the trial side and re-anastomosed. Flaps and anastomoses were assessed before, during, and after the period of ischemia every ten minutes for overall 60 min using HSI and MDS. The contralateral sides’ flaps served as controls. Tissue-oxygenation saturation (StO_2_), near-infrared perfusion index (NPI), hemoglobin (THI), and water distribution (TWI) were assessed by HSI, while blood flow was assessed by MDS. HSI correlates with the MDS signal in the case of sufficient and completely interrupted perfusion. HSI was able to validly and reproducibly detect tissue perfusion status using StO_2_ and NPI. After 40 min, flap perfusion decreased due to the general aggravation of hemodynamic circulatory situation, which resulted in a significant drop of StO_2_ (*p* < 0.005) and NPI (*p* < 0.005), whereas the Doppler signal remained unchanged. In accordance, HSI might be suitable to detect MFF general complications in an early stage and further decrease MFF failure rates, whereas MDS may only be used for direct complications at the anastomose site.

## 1. Introduction

Microvascular free flap (MFF) transfer is one of the most common methods in head and neck reconstructive surgery to reconstruct defects after tumor surgeries, accidental and blast injuries, or burns. Depending on the type of defect and the requirements of the patient, various types of free flaps are available. The radial or ulnar forearm flap, the anterolateral thigh (ALT) flap, the upper arm flap, or the latissimus dorsi flap are available for soft-tissue reconstructions. If bony reconstruction is required, osteomyocutaneous fibula free flap, scapula flap, or deep circumflex iliac artery (DCIA) flap can be used. Depending on the type of flap, there is a flap survival rate of over 95% [1,2,3,4,5]. In addition to the type of MFF and the surgeon’s experience, several factors influencing the success rate of free tissue transfer have been identified. These include patient-dependent and patient-independent factors, among others, duration of surgery over 8 h, need for intraoperative re-anastomosis, anatomical complexity, difficult micro-anastomoses, or arterial and venous thrombosis [6,7]. Free flap failure is a dynamic and often multifactorial process that ranges from a reversible complication to complete flap failure [8]. In addition to the loss itself and thus the lack of reconstruction, a transplant failure leads to further disadvantages. These include a decreased overall patient outcome, delayed wound healing and thus a later start of adjuvant radiotherapy or chemotherapy, reduced opportunities for secondary reconstruction, the need for additional anesthetics and surgery, longer hospital stays, and not least of all a financial burden on the healthcare system [9,10]. Flap failure can usually be caused by vascular complications, bleeding, hematoma, fistula, or infection. Although venous insufficiency is the most common complication of MFF, arterial perfusion disorders are the main reasons for flap failure due to poorer salvage rate [11,12]. Therefore, adequate perfusion monitoring is extremely important to detect these vascular problems as early as possible and to intervene before irreversible damage has occurred. To avoid a partial or complete loss of the transplant, it should be revised without delay because early interventions have a higher chance of transplant salvage [7]. According to Chao et al., the perfect monitoring method should be inter alia precise, objective, reproducible, easy to interpret, non-invasive, and safe [13]. Several non-invasive and invasive monitoring methods, such as different Doppler sonography methods, near-infrared spectroscopy, microdialysis, tissue oximetry, and even digital photography using smartphones, have been established for this purpose although the clinical assessment of MFF is still the gold standard [14,15,16]. A major disadvantage of clinical assessment is the strong intra- and inter-individual examiner dependency. In addition, experience is required to be able to make a precise statement about the flap situation. However, since experienced medical specialists cannot be available round the clock, valid device-based monitoring methods are preferable to the less experienced resident or nurse in terms of objectivity and interpretation of the clinical flap status [1]. In this context, hyperspectral imaging (HSI) is a non-contact imaging technique for medical applications. It was developed for the determination of tissue perfusion and oxygenation and has been used for the evaluation of diabetic foot, burns, skin ulcer, wound analysis, and flap monitoring [17,18,19,20,21], technically based on the optical evaluation of light spectra in the visible and near-infrared range, which are remitted by individual tissue molecules. The detection of water, oxyhemoglobin, and deoxyhemoglobin allows conclusions to be drawn about the oxygen supply and blood flow in the tissue. HSI offers some properties of an ideal monitoring method. As an investigator-independent, non-invasive, non-ionizing method that is safe for the patient and examiner, it can precisely, objectively, and reproducibly measure various parameters of tissue perfusion and oxygenation and could therefore represent a clinical monitoring method for intra- and postoperative microvascular free flap assessment [1,22,23]. An established standard method for postoperative assessment of anastomoses is monitoring using implantable micro-Doppler sonography (Cook–Swartz Doppler) [24]. Several meta-analyses have shown that postoperative monitoring using Cook–Swartz Doppler can achieve a higher free flap and salvage success rate when compared to clinical assessment. A disadvantage can be a higher false-positive rate, which leads to unnecessary interventions [25,26]. Moreover, it is also an invasive monitoring method. In this context, cases of material failure with necessary second interventions during probe removal have been reported [27]. In addition, micro-Doppler sonography is a widely used standard method for intraoperative hemodynamic assessment in vascular surgery [28,29]. The authors are not aware of any study that quantitatively compares HSI with another device-based monitoring method in the field of microvascular free flap pre-, intra-, and postoperative monitoring in a standardized, in vivo rodent model/study design. In previous studies, clinical assessment or hand-held Doppler-assisted clinical assessment served as a reference method for MFF assessment by HSI [6,30]. In this study, absolute measurement parameters of the HSI should be compared and correlated with absolute measurement parameters of the MDS since no HSI cut-off values for a free flap failure exist so far. The main difference between the two methods is that the HSI measures the area of the capillary terminal flow of the MFF, while MDS assesses the perfusion in the area of a comparatively large lumen supply vessel directly distal to the anastomosis. The aim of this study was to evaluate HSI in comparison to MDS to monitor MFF and anastomoses in an in vivo model in rodents.

## 2. Materials and Methods

### 2.1. Surgery

**Figure 1:**
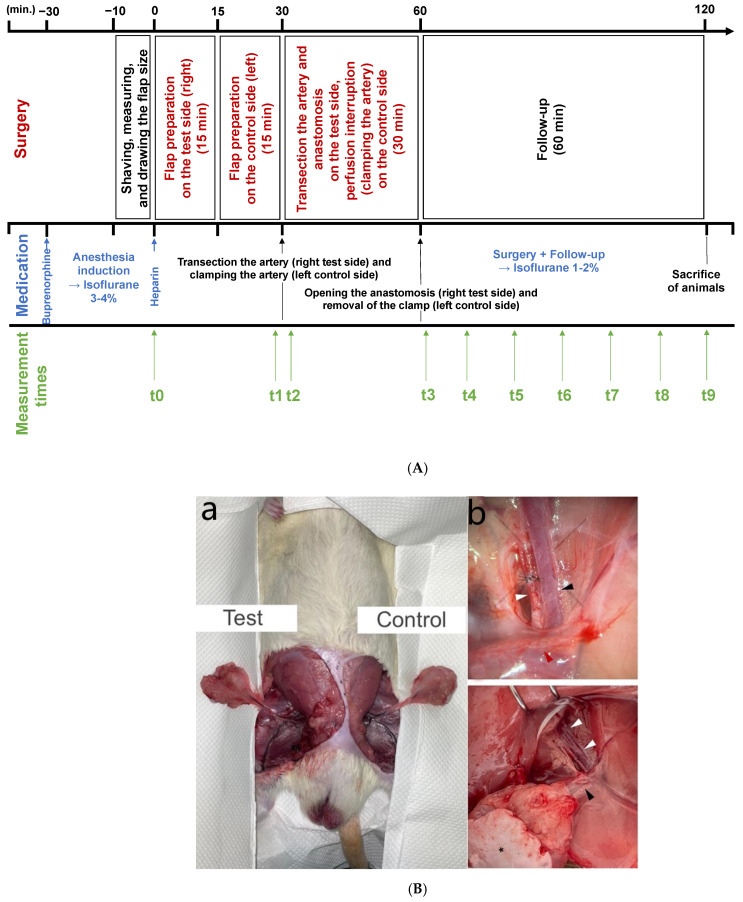
(**A**) Project timeline showing anesthesia, surgical steps, and measurement timepoints. (**B**) Illustration of the experimental setup with (a) bilateral dissection of the groin flap and ((b), top picture) overview about the arterial anastomoses. The femoral artery (white arrow) is anastomosed by 10.0 sutures. The femoral vein (black arrow) is illustrated directly below. The red arrow highlights the superficial epigastric vessels. ((b), bottom picture) Overview of the adipocutaneous groin flap on the left side of the rat. The femoral vessels (white arrows) and the superficial epigastric vessels (black arrows) create the flap pedicle, artery, and vein, respectively. The flap consists of a skin isle (black asterisk) and the underlying subcutaneous fatty tissue. For detailed anatomical information, see Wallmichrath et al. [31].

Sprague–Dawley rats (n = 20, male, ±12 weeks old, weighing each between 300–500 g) were kept under standard conditions (12 h day/night, 20 °C, humidity 50%), chow and water were ad libitum, and the animals were under constant observation. All experiments were approved by the animal protection authority of the state Rhineland-Palatinate (Koblenz, Germany, no. G 19-1-078) and in accordance with the ARRIVE guidelines. Before surgery, animals were first weighed. They were put under anesthesia starting with an injection of 0.1 mg/kg body weight (b.w.) buprenorphine intraperitoneally (i.p.) (Temgesic^®^ 0.3 mg/mL; Indivior Europe Limited, Dublin, Ireland) 30 min (min) before surgery. Then, animals were put in an anesthesia box with 3–4% isoflurane (isofluorane 99.9%; Provet AG, Lyssach b. Burgdorf, Switzerland), which was then reduced to 1–2% isoflurane via mask for maintenance (after effect). During surgery, animals received additional oxygen via a mask with simultaneous peripheral ex vivo monitoring of oxygen saturation. For perioperative anticoagulation, animals were administered subcutaneously with unfractionated heparin (heparin-natrium-ratiopharm, 5000 IU/0.2 mL, ratiopharm GmbH, Ulm Germany) in a dose of 100 IU/kg b.w., and the wound area was also rinsed during the surgery of the anastomosis with a heparinized lavage (10 IU/mL). After checking the interdigital reflex until adequate pain elimination was achieved, shaving and repeated skin disinfection were performed, and groin flaps [31] were raised on both sides after drawing in the flap size (medial-to-lateral 3 cm and caudal-to-cranial 4 cm) (Figure 1A). The dissection of groin MFF in rats was first described in 1967 by Strauch and Murray [32]. It is associated with little stress on the animals and has been investigated in numerous studies on MFF hemodynamics [31,33,34]. Beginning on the right test side and starting from the femoral artery and vein, the superficial epigastric artery and vein were sought, and a spindle-shaped adipocutaneous flap, which is supplied exclusively by superficial epigastric vessels, was prepared. Hence, following ligation and transection of the femoral vessels, malperfusion of the groin flap was revealed then returned to normal after re-anastomosis. The femoral artery was surgically exposed at their outlet from the iliac artery. In addition, a temporary ligation of the femoral artery distal to the outlet of the superficial epigastric artery was necessary to avoid flow reversal in the femoral artery and thus retrograde supply of the groin flap from collateral circulation. Re-perfusion was achieved by microvascular suture (Ethilon™ 10-0, Ethicon, Inc., Raritan, NJ, USA). Next, flap preparation was performed in the same way on the contralateral left control side. However, instead of transection, blood flow was stopped by temporary clamping. Perfusion monitoring was performed for 60 min after re-perfusion using hyperspectral imaging (HSI; TIVITA™, Diaspective Vision, Pepelow, Germany) and micro-Doppler sonography (MDS; Compumedics Germany GmbH, Singen, Germany) (Figure 1A,B). Perioperative vasospasms were treated topically with papaverine hydrochloride (1 mg/mL) (Papaveron N injection solution, 25 mg/mL, LINDEN arzneimittel-vertrieb-GmbH, Heuschelheim, Germany) by sprinkling the vascular segment. Due to the long duration, the surgery was carried out using a heat mat and constant monitoring of the body temperature. After one hour of observation time after anastomoses, the monitoring was terminated, and the animals were sacrificed through exsanguination under sufficient anesthesia.

### 2.2. Hyperspectral Imaging

For hyperspectral imaging, the camera system TIVITA™ Tissue System was used. It essentially consists of a hyperspectral camera, a lens, and a light source consisting of six halogen lamps (20 W each) arranged around the hyperspectral camera. The camera system is attached to a swivel arm that can be adjusted in height and direction. There is also a box computer and the processing software TIVITA™ Suite. The measuring distance is usually 50 cm. This is achieved by setting points of light in an overlapping position. The system captures a three-dimensional HyperCube in which the first and second dimensions represent the spatial information (resolution: 0.1 mm/pixel at a distance of 50 cm). The spectral information (resolution: 5 nm) is recorded in the third dimension [35]. The measurements are based on the evaluation of light spectra in the wavelength range from 500 nm to 1000 nm, which are remitted by individual tissue molecules after exposure to the halogen light sources. These measured wave spectra are molecule-specific, allowing the system to detect the molecules of interest: oxyhemoglobin, deoxyhemoglobin, and water [36]. From this, the algorithm calculates four false-color images that represent the parameters tissue-oxygenation saturation (superficial perfusion at a depth of 0–1 mm; StO_2_ (0–100%)), near-infrared perfusion index (deep perfusion at a depth of 0–6 mm; NPI (0–100)), hemoglobin distribution (THI; (0–100)), and water (tissue water index; TWI (0–100)). In addition, a true color image (red, green, and blue) is recorded. The superficial perfusion StO_2_ indicates the hemoglobin oxygen saturation percentage in the capillary area of the tissue. Tissue oxygen saturation includes arterial and venous blood and detects changes in the tissue oxygen supply. It is largely based on the venous part (75%) of the microcirculation and its oxygen saturation after oxygen delivery to the surrounding tissue [1,37]. Although there are no uniform standard values to date, the StO_2_ values in healthy people are usually around 50–70% [38]. The NPI perfusion parameter describes the quality of the blood flow. This is determined on the one hand by the relative hemoglobin content and on the other hand by the relative oxygen saturation of the hemoglobin in the capillary circulatory system in tissue layers of 4 to 6 mm. NPI perfusion shows tissue areas with reduced blood flow in deeper tissue layers. THI (tissue hemoglobin index) describes the superficial hemoglobin distribution in the microcirculation of the superficial tissue. With this index parameter, in combination with StO_2_ or NPI, arterial supply or venous outflow problems can be recognized and differentiated. As a further index parameter, the TWI describes the relative water content in the tissue area under consideration [1,22,35,36,37]. For the evaluation and quantitative determination of the four parameters, a circular region of interest (ROI; 100 pixel), which contains the mean value of the spectral and spatial information per pixel, was manually positioned on the images, always in the same flap area. The software automatically calculated the mean values within the ROI for each perfusion parameter (StO_2_, NPI, THI, and TWI). The measurement times were determined as follows: after shaving and before lifting the flap (t0), after lifting the flap (t1), after transection or after clamping the artery (t2), after opening the anastomosis (t3), and after 10 (t4), 20 (t5), 30 (t6), 40 (t7), 50 (t8), and 60 min (t9) (Figure 1).

### 2.3. Micro-Doppler Sonographic Measurement

For the micro-Doppler sonographic measurement, the DWL Doppler-BoxX 1 system (Compumedics Germany GmbH, Singen, Germany), which was connected to an external tablet computer with the associated DWL software, was used with a 16 MHz microprobe (Ø 1.8 mm). The sensitivity settings were made according to the manufacturer’s recommendations (sample volume 7, sensitivity 50%, performance 84, depth 0.8 mm, filter 50, angle correction 0). The angle of the probe to the artery was set at 45–60°. Micro-Doppler sonography is based on the Doppler effect. This describes how the frequency of a sound signal shifts when it hits an object that is moving relative to it. The frequency shift to be measured depends, among other things, on the speed of the moving object: in this case, on the flow velocity of the corpuscular components of the blood, which the Doppler signal encounters. According to the following formula, the frequency shift ∆F is related to the transmission frequency F_0_, the speed of sound in blood c (c_blood_= 1570 ms^−1^ [39]), and the blood flow velocity v [40,41]:

$$\Delta F=\frac{2\times F_{0}\times v}{c}$$

Thus, by rearranging the formula and measuring the frequency shift, the blood flow velocity v can be calculated:

$$v=\frac{\Delta F\times c}{2\times F_{0}}$$

Once the flow velocity v has been calculated, the blood volume flow V can be calculated using the following formula if the vessel diameter d (d_fem-rat_ = 0.54 mm [42]) is known [43]:

$$V=v\times \frac{\Pi }{4}\times d^{2}$$

**Figure 2:**
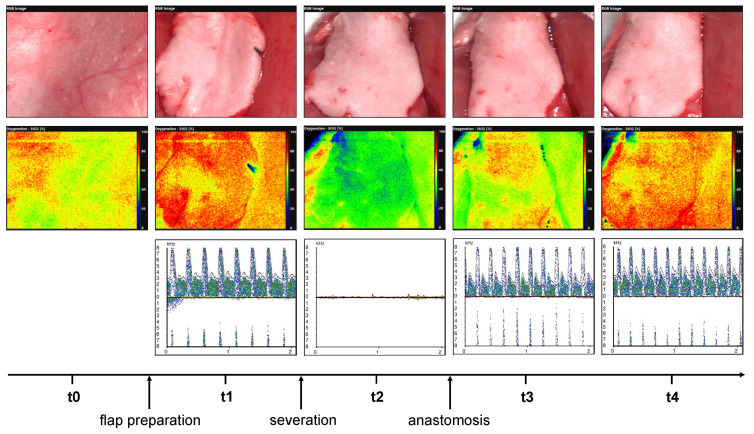
Illustrates hyperspectral analysis at time points t0, t1, t2, t3, and t4 (middle row) and micro-Doppler analysis at time points t1, t2, t3, and t4 (lower row). The respective clinical pictures are shown in the upper row.

After flap preparation, the mean frequency shifts were determined at the specified measurement times using Doppler, and the respective volume flow rates were calculated and compared. The measurement times correspond to those of the hyperspectral measurements (Figure 2).

### 2.4. Statistics

The primary parameters, namely tissue oxygenation saturation (StO_2_), near perfusion index (NPI), tissue hemoglobin index (THI), and tissue water index (TWI) were assessed as primary parameters using HSI as well as doppler flow characteristics, including maximum doppler frequency (Max.KHz), mean doppler frequency (Mean.KHz), resistance index (RI), and pulsatility index (PI), using MDS. Raw data sets were saved in Excel^®^ sheets (Microsoft Corporation, Redmond, WA, USA) and subsequently transferred into SPSS Statistics^®^ (version 23.0.0.2, MacOS X; SPSS Inc., IBM Corporation, Armonk, NY, USA). Data are expressed as mean (m), standard deviation (SD±), minimum (min), and maximum (max). Normal distribution was checked using non-parametric Shapiro–Wilk test. Qualitative variables were summarized as counts and percentages. In addition to the descriptive analysis, the dependency analysis included tests to detect/exclude differences and correlations. Results were analyzed for statistical significance by the use of analysis of variance (ANOVA with repeated measures), unpaired non-parametric Mann–Whitney U-tests and Student’s *t*-test. To investigate whether the means of several dependent samples differ, Wilcoxon matched-pairs signed rank test was performed. Correlations between two categorical variables were tested using the Pearson’s chi-square test or, in the case of expected cell frequencies < 5, using Fisher’s exact test. *p*-values of ≤ 0.05 were termed significant. Line charts with plotted means, dot plots, and bar charts were used for illustration purposes. Since the present study is a first description of the methodology on the animal model used, no power calculation could be performed.

## 3. Results

### 3.1. Hyperspectral Perfusion Analysis

#### 3.1.1. Tissue Oxygenation Saturation (StO_2_)

**Figure 3:**
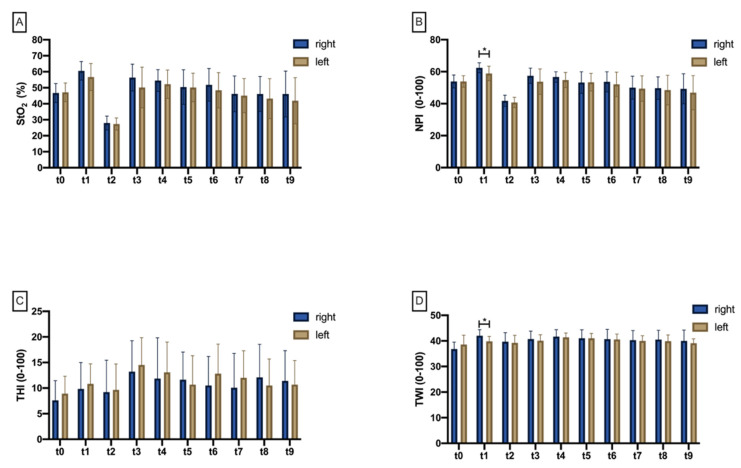
Bar chart with means (±SD) showing (**A**) tissue oxygenation saturation (StO_2_), (**B**) near-infrared perfusion index (NPI), (**C**) tissue hemoglobin index (THI), and (**D**) tissue water index (TWI) at different measurement timepoints (t1–t9). Crossbars with asterisks mark significant differences.

**Figure 4:**
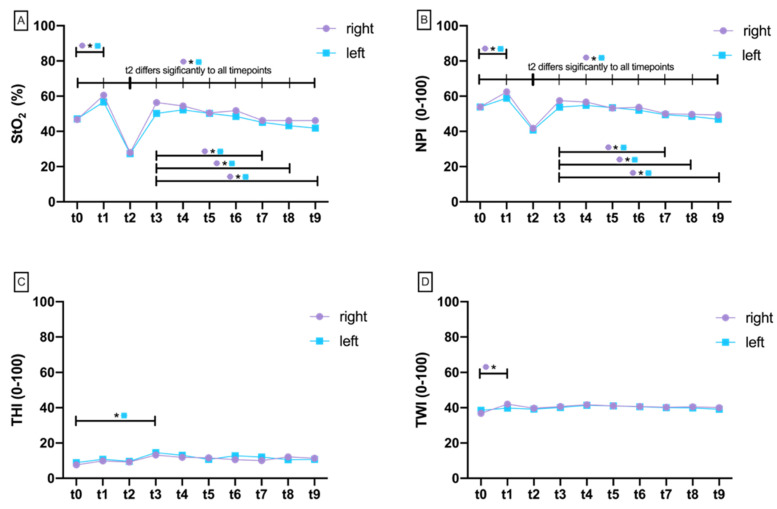
Aligned dotplot show hyperspectral parameters at different measurement timepoints (t0–t9) as mean values. (**A**) Tissue oxygenation saturation (StO_2_), (**B**) near-infrared perfusion index (NPI), (**C**) tissue hemoglobin index (THI), and (**D**) tissue water index (TWI). Crossbars with asterisks mark significant differences.

The Shapiro–Wilk test failed to reject the 0 hypothesis of a normal distribution at all ten measurement time points. There was no significant difference of StO_2_ at any time point when comparing test (right) and control (left) sides (Figure 3A, Table S1). There was a significant difference at the test side over time between baseline (t0) and flap preparation (t1) (*p* = 0.006) as well as between t0 and vessel transection/clamping (t2), showing a significant StO_2_ drop (*p* < 0.001; Figure 4A). After re-perfusion (t3), there was a significant increase in StO_2_ on both the test and control sides, which was bilaterally significant until t9 (40 min after re-prefusion) compared with t2 (*p* < 0.001; Figure 4A). From 40 min after re-perfusion (t7), StO_2_ significantly dropped below re-perfusion status (t3) on both sides (*p* = 0.010; *p* = 0.003), which was also revealed as significant for t8 (*p* = 0.014; *p* = 0.003) and t9 (*p* = 0.022; *p* = 0.004) (Table S1; Figure 4A).

#### 3.1.2. Near Perfusion Index (NPI)

The Shapiro–Wilk test was unable to reject the 0 hypothesis of a normal distribution at all ten measurement time points. When comparing the right test and the left control side, the near-infrared perfusion index was significantly reduced exclusively at t1 (after flap preparation) on the left side (*p* = 0.004; Figure 3B). Over time, both right (*p* < 0.001) and left (*p* = 0.010) sides revealed a significant increase in NPI (Figure 4B). With perfusion breakdown (t2), there was a significant drop in NPI bilaterally (*p* < 0.001). The subsequent increase after re-prefusion was significant on both sides up to t9 (*p* < 0.05; Figure 4B). From 40 min on (t7), NPI dropped significantly below re-prefusion status (t3) on both sides (*p* = 0.011; *p* < 0.001), which was revealed as significant for t8 (*p* = 0.008; *p* = 0.002) and t9 (*p* = 0.017; *p* = 0.003) (Table S1; Figure 4B).

#### 3.1.3. Tissue Hemoglobin Index (THI)

The Shapiro–Wilk test was unable to reject the 0 hypothesis of a normal distribution at all ten measurement time points. There was no significant difference at any measurement time point comparing right test and left control sides (Table S1, Figure 3C). THI revealed no significant changes from baseline (t0) to vessel transection/clamping (t2). Only at t3 was there a significant increase on the left side (*p* = 0.013). In the further course until t9, there were minor changes on both sides without return to baseline level (Table S1, Figure 4C).

#### 3.1.4. Tissue Water Index (TWI)

According to the Shapiro–Wilk test, data were normally distributed at all time points. Comparing the right test and left control sides, there was a significant difference at t1 with increased TWI on the right side (*p* < 0.001; Table S1; Figure 3D). At subsequent measurement time points, there was no difference between both sides. Between measurement time points, the only difference was found between t0 and t1, with significantly increased TWI on the right side (*p* = 0.004; Table S1; Figure 4D).

### 3.2. Micro-Doppler Sonographic Measurement

#### 3.2.1. Maximum Doppler Frequency (Max.KHz)

**Figure 5:**
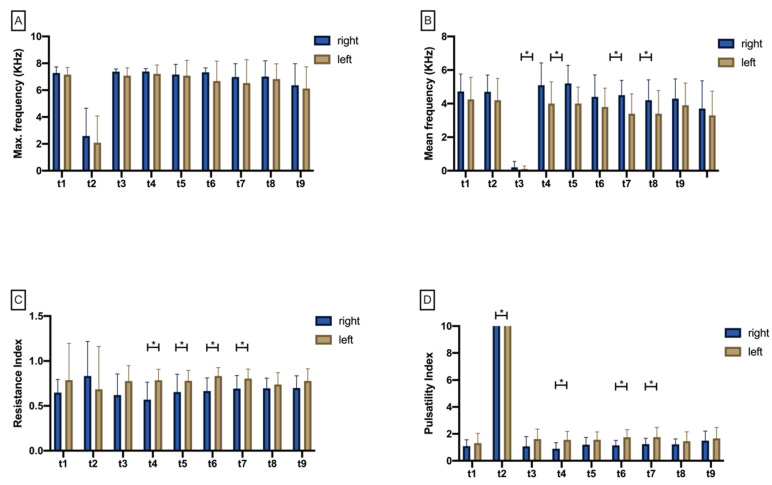
Bar chart with means (±SD) showing Max.KHz (**A**), Mean.KHz (**B**), resistance index (**C**), and pulsatility index (**D**) at different measurement timepoints (t1–t9). Crossbars with asterisks mark significant differences.

**Figure 6:**
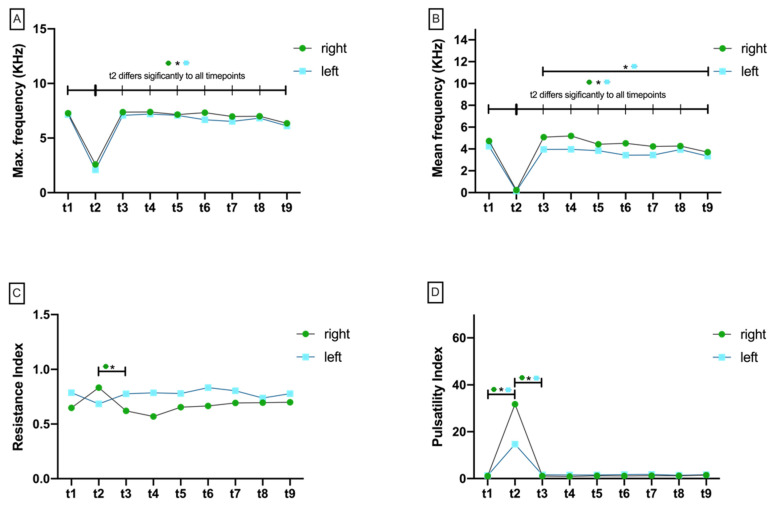
Aligned dotplot show doppler characteristics at different measurement timepoints (t1–t9). (**A**) Maximum frequency in KHz (Max.KHz), (**B**) Mean frequency in KHz (Mean.KHz), (**C**) resistance index (RI), and (**D**) pulsatility index (PI). Crossbars with asterisks mark significant differences.

The Shapiro–Wilk test rejected the null hypothesis of a normal distribution for all ten time points (*p* < 0.05). There was no significant difference of Max.KHz at any time point when comparing test (right) and control (left) side (Figure 5A, Table S2). After flap preparation (t1), there was a significant drop in Max.KHz on both the right (*p* = 0.004) and the left (*p* = 0.005) sides as a result of perfusion breakdown (t2) (Table S2; Figure 6A). After perfusion was restored, Max.KHz increased significantly back toward baseline on both the right and the left sides (*p* = 0.004, *p* = 0.005). During the following measurement points, Max.KHz remained consistently high until 60 min after re-perfusion (t9) without significant changes from t1 (Table S2; Figure 6A).

#### 3.2.2. Mean Doppler Frequency (Mean.KHz)

The Shapiro–Wilk test rejected the null hypothesis of a normal distribution for all ten time points (*p* < 0.05). After re-perfusion (t3), Mean.KHz was significantly reduced on the left side compared with the right test side (*p* = 0.039). Mean.KHz was also found to be increased on the right test side at time points t4 (*p* = 0.023), t6 (*p* = 0.003), and t7 (*p* = 0.008) (Figure 5B). After flap preparation (t1), there was a significant drop in Mean.KHz on both sides (*p* < 0.001) as a result of perfusion breakdown (t2). After re-prefusion, Mean.KHz significantly increased back toward baseline values on both sides (*p* < 0.001). In the further course of the experiment, there were non-significant changes in Mean.KHz on both sides compared with t1 until the end of the experiment after 60 min (t9) (Table S2; Figure 6B).

#### 3.2.3. Resistance Index (RI)

The Shapiro–Wilk test rejected the null hypothesis of a normal distribution for all ten time points (*p* < 0.05). The resistance index was found to be only slightly different between right test and left control side up to and including t3 (re-perfusion). In contrast, RI was more significantly increased on the left side than on the right test side at t4 (*p* = 0.009), t5 (*p* = 0.037), t6 (*p* = 0.005), and t7 (*p* = 0.026) (Table S2; Figure 5C). Over time, RI was found on both sides without significant differences from t1 to t9 (Table S2; Figure 6C).

#### 3.2.4. Pulsatility Index (PI)

The Shapiro–Wilk test rejected the null hypothesis of a normal distribution for all ten time points (*p* < 0.05). The pulsatility index (PI) was insignificantly different comparing right and left sides from t1 to t3. Ten, thirty, and forty minutes after reperfusion, PI was significantly increased (*p* = 0.023; *p* = 0.002; *p* = 0.008) on the left control side compared with the right test side (Table S2; Figure 5D). Over time, PI was found on both sides without significant differences from t3 to t9 (Table S2; Figure 6D). However, following perfusion breakdown (t2), PI significantly increased on both the right test and the left control side (Table S2; Figure 6D).

#### 3.2.5. Variance between Max.KHz and Mean.KHz

**Figure 7:**
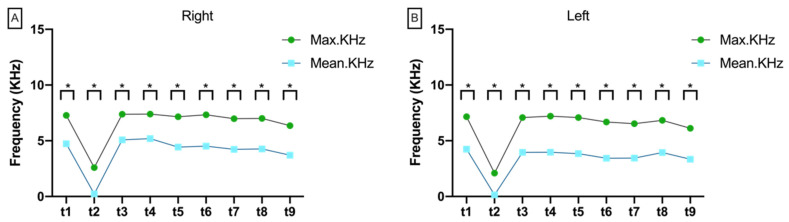
Aligned dotplot shows maximum Doppler frequency (Max.KHz) and mean Doppler frequency (Mean.KHz) at the measurement times t1 to t9 on the (**A**) right and (**B**) left side. Asterisks indicate significant differences between Max.KHz and Mean.KHz.

The variance between maximum Doppler frequency (Max.KHz) and mean Doppler frequency (Mean.KHz) was significant on both sides at each measurement time point (*p* < 0.008; Figure 7). Overall, there was a constant increase in the difference between Max.Khz and Mean.KHz on both the right and left sides (Table S3).

### 3.3. Comparison of HSI and MDS

**Figure 8:**
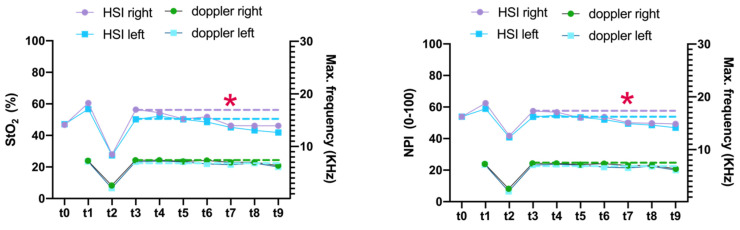
Aligned dotplots illustrate the lack of change compared to the hyperspectral parameters StO_2_ and NPI on the example of the maximum Doppler frequency (Max.frequency KHz). The red asterisk indicates onset of significant decreases in StO_2_ and NPI at t7. The dashed lines indicate the parameter value level at t3.

**Figure 9:**
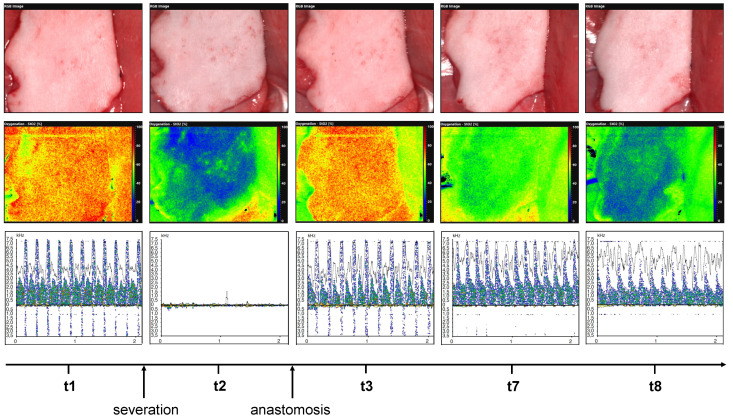
Illustrates hyperspectral analysis (middle row) and micro-Doppler analysis (lower row) at time points t1, t2, t3, t7, and t8 of an example of groin flap with onset of malperfusion from t7. The respective clinical pictures are shown in the upper row.

Overall, the hyperspectral microcirculation parameters StO_2_ and NPI significantly decreased from 40 min after reperfusion (t3) up to and including t9. There were no corresponding changes in the various Doppler parameters (Max.KHz, Mean.KHz, RI, and PI) from t3 to t9 (Figure 8). To illustrate this phenomenon, Figure 9 exemplarily compares HSI and MDS measurements on the example of an insufficiently perfused groin flap.

## 4. Discussion

This study compared HSI and MDS for perfusion monitoring of groin MFF and anastomoses in an in vivo model in rodents. We demonstrated that hyperspectral imaging is a valid and reproducible method for flap monitoring, which detects relevant microvascular perfusion changes earlier than micro-Doppler sonography. As early as in 1986, the rat groin flap model showed that MDS can be a promising method for the perioperative evaluation of anastomoses and for the postoperative monitoring of MFF, with flow velocity values similar to this study being measured in the area of the femoral artery distal to the anastomosis [44]. A major advantage of the rat groin flap model is that a complete perfusion interruption of the MFF can be achieved by adequate clamping or transection of the femoral artery, which could be confirmed by both HSI and MDS in this study. Indeed, the alternative hemodynamic animal model of hind limb ischemia is accompanied by even less traumatization of the tissue and thus less stress on the animal, but hind limb ischemia cannot always be ensured by transecting the femoral artery alone since there is usually a collateral supply to the hind limb [45,46]. On the other hand, an assessment of tissue perfusion after anastomosis of the femoral artery was also successfully demonstrated in the rat hind limb model exclusively using HSI [23]. Other advantages of the groin flaps are that they can be carried out safely, quickly, and reproducibly. Vessel preparation is easy and is associated with little trauma to the animal, as their diameter is large enough for anastomosis with suture. In contrast to this experiment, even a complete removal of the flap, including the vascular pedicle with transplantation to the neck or into the mouth of the rat, can be performed, which, however, is associated with a higher complication rate due to a greater tissue trauma and a longer duration of surgery [47]. This was not necessary for the aim of this study and was therefore not carried out. Although many studies have been able to highlight an advantage for device-based monitoring methods compared to clinical assessment, and these are commercially available, they are only rarely used. This is probably due to the high acquisition or follow-up costs of some device-based monitoring methods. In addition, however, some device-based monitoring methods and the measured values also depend on expert interpretation [48]. Indeed, HSI implementation is associated with high prices, but after a one-time purchase, there are no further follow-up costs, e.g., for disposable probes, and could pay off if the length of hospital stay is shortened through earlier detection of arterial perfusion disorders and more timely salvage surgeries, thereby reducing the overall MFF failure rate [49,50].

In the present study, flap perfusion revealed similar values in a side-by-side comparison during HSI analysis (StO_2_, NPI, THI, and TWI). Although not significant, the direct perfusion parameters StO_2_ and NPI revealed increases on average at t4, t5, t6, t7, t8, and t9 on the right test side compared with the left control side after flap reperfusion (t3). Considering parameter dynamics in this context, there was a significant decrease in StO_2_ and NPI after perfusion breakdown (t2) as well as a subsequent significant rebound after re-perfusion on both sides (t3). For micro-Doppler-scanning, the maximum Doppler frequency (Max.KHz) also revealed no difference between the right and the left side. In contrast, Mean.KHz was significantly increased at t3, t4, t6, and t7 on the right test side compared to the left control side. However, mean Doppler frequency can only be used in conjunction with the maximum Doppler frequency (i.e., the difference between Max.KHz and Mean.KHz) to provide information on the severity of a vascular stenosis. The difference between Max.KHz and Mean.KHz on the right side after re-perfusion (t3) was 4% below baseline (t1), indicating increased blood flow. On the left control side, there was a 2.3% increase in the difference after release of the vascular clamp (t3), indicating a reduction in perfusion. Overall, both sides revealed a constant increase in Max.–Mean.KHz difference until t9, which also indicates a reduction of vascular blood flow (Figure 7; Table S3). However, lack of reference data makes data interpretation impossible. The resistance index (RI) according to Pourcelot allows conclusions about the arterial flow resistance. On examination of a muscle-supplying artery, values around 1 indicate increased vascular tone, whereas values below that indicate post-stenotic vasodilation. Micro-Doppler sonography (MDS) allows examination and monitoring of vascular blood flow in real-time, whereby resistance index (RI) is < 0.70 in average, even in rodents [51,52]. Additionally, in our study, the mean RI from t1 to t9 was 0.7 ±0.2. Absolute pulsatility is difficult to assess by Doppler sonography because the amplitude of the pulsatile blood flow velocity is dependent on the angle of insonation. Thus, several angle-independent pulsatility indices were defined, of which the Gosling index of pulsatility (PI) became the most popular. PI was defined as the difference of peak systolic and lowest diastolic flow velocities referenced to time-averaged flow velocity [53]. The interference signal is derived distally, i.e., in the direction of flow behind the stenosis, whereby the distance between stenosis and derivation position is not relevant. Except for t2 (condition after vessel transection/clamping), the PI on the right test side in our study was 1.2 ± 0.5 and on the left control side 1.6 ± 0.7. At t2, the PI on the right (31.8 ± 32.4) and left side (14.7 ± 21.6) was very high, most likely due to the lack of blood flow. Overall, the validity of the pulsatility index is limited, as related studies show controversial data in rodents ranging between 4.7 for the right carotid and 1 for the femoral artery as baseline values in mice. In the present study, there was a significant decrease in hyperspectral tissue oxygen saturation (StO_2_) and near-infrared perfusion index from t7, whereas there were no significant changes in doppler characteristics (illustrated by maximum Doppler frequency).

A systematic review examined HSI compared to near-infrared spectroscopy (NIRS) as monitoring methods for the detection flap failure and was unable to determine any superiority of one of the two methods [49]. The authors concluded that both methods are valid for detection of MFF failure postoperatively but cannot replace the gold standard of clinical assessment to date. In two recently published prospective clinical studies, it was shown that HSI can detect perfusion problems much earlier than (hand-held Doppler-assisted) clinical assessment [6,30]. Perhaps the most commonly used device-based MFF monitoring method is the implantable MDS. It is considered to be simple to use and to interpret, safe, examiner-independent, efficient, and can also be used successfully with buried flaps. The invasiveness, a possible focus of infection, and the risk of injury when removing the sample are regarded as disadvantages [50,54,55]. In addition, it also seems that complete MFF necrosis can occur despite an adequate MDS signal and thus despite adequate blood flow through the anastomosis [55]. It was also observed in this study that MDS showed an unchanged Doppler signal, while HSI had demonstrated a non-perfused flap. A possible explanation may be that the cause of the perfusion problem was further distal to the measuring point and was not recorded by MDS. At this point, there is a clear advantage in monitoring methods that measure the area of the capillary terminal flow path. Nevertheless, implantable MDS was able to assert itself against microdialysis, clinical assessment, and external hand-held Doppler [54,55]. To the authors’ knowledge, no study has compared HSI to MDS, but there is preclinical and clinical research on monitoring flaps using HSI. Chin et al. were able to show that early changes in HSI imaging correlate with later random pattern flap necrosis in a rodent model [56]. In a similar study by Müller-Seubert et al., however, this was not confirmed. In addition, HSI was inferior to indocyanine green angiography (ICG) angiography in predicting necrotic areas in rat random pattern flaps. Furthermore, evaluation of the flaps by HSI was not even possible in the study, but this may have been due to the experimental design rather than the HSI technique itself [57]. In contrast, Thiem et al. [1] used HSI successfully for perioperative monitoring of free and pedicled flaps. However, it must be noted that a distinction has to be made between random pattern flaps and free/pedicled flaps, which can be assigned to definitive vessels. However, monitoring using HSI is probably also possible with random pattern flaps, which should be confirmed by future studies. In this study, possible cut-off values for the need for flap revision (StO_2_ < 45% and NPI < 25/100) and the distinction between venous and arterial problems were also described for the first time [1]. Kohler et al. [30] hypothesized similar cut-off values (StO_2_ < 40% and NPI < 40/100), whereas in a recent study by Thiem et al., the StO_2_ and/or NPI drop rate as a relative to a reference value was preferred instead of absolute cut-off values since absolute values do not consider the dependency of StO_2_ and NPI on the systemic Hb concentration. Based on 100 patients, Heimes et al. described mean StO_2_ of 40% after palmar perfusion breakdown. In addition to these absolute values, a relative before–after value was also reliably used to assess perfusion [37].

Despite the advantages of HSI, such as objectivity, reproducibility, examiner independence, and non-invasiveness, there are also disadvantages and issues that still need to be clarified. One disadvantage of the hyperspectral camera system used here is the inability to position the camera itself intraorally on the one hand because of the size and on the other hand because of the measuring distance of 50 cm. It is therefore not always possible to obtain a meaningful measurement in the case of exclusively intraoral reconstructions, such as in the dorsal floor of the mouth, at the base of the tongue, or in the area of the soft palate. This problem could be solved in the future by a newly developed endoscope HSI system [6]. In addition, there is no research on HSI monitoring of buried flaps so far. In the case of MFFs that do not have a skin island and are completely covered by local tissue, HSI monitoring initially seems theoretically unsuitable. With the optical method of NIRS, monitoring of buried flaps is possible if the tissue layer is not thicker than the maximum depth range of the sensor. It can therefore be assumed that buried MFFs could be monitored if they are covered by tissue with a thickness of less than 5 mm, which would represent a clear advantage over clinical assessment since buried flaps obviously cannot be assessed clinically. Furthermore, it should be investigated to what extent the anastomosis or the flap pedicle itself could be assessed intraoperatively or if the direct detection of stenoses is possible using HSI, as can be successfully carried out with ICG angiography and MDS [28]. A clear limitation of the study is that only the artery was anastomosed although venous insufficiency is the most frequent complication associated with MFF surgery. In this study, however, only the artery should initially be addressed for perfusion monitoring since arterial problems are the main cause of complete MFF failure [11,12]. The advantage here is that a clear correlation between MDS and HSI was possible. If both the artery and the vein had been anastomosed, there would have been two measuring points for MDS (distal to the arterial and distal to the venous anastomosis) but still only one measuring point for HSI, which makes a clear comparison of the two methods difficult and may reduce the significance. On the other hand, by a combination of the parameters StO2/NPI and TWI/THI, it might have been possible to differentiate between the arterial and venous situation using HIS [1]. In addition, the anastomosis of both vessels significantly increases the duration of surgery and anesthesia and thus also stress on the animals, which has led to a high drop-out rate in other rat groin flap studies [47]. It is desirable that follow-up trials also address the vein, perhaps in a bi-vessel model that most closely matches the patient’s situation in reconstructive MFF surgery. A possible limitation of HSI is that there is no continuous measurement, such as with implantable MDS or NIRS. However, this could possibly be circumvented by combining HSI with other monitoring methods or shortening the HSI measurement intervals for MFF that have a high-risk profile. To enable frequent measurements, staff must be trained to operate the HSI device correctly and to interpret the values safely. For this, it will be necessary either to generate cut-off values or to define drop rates (e.g., decrease from baseline), which are recorded in standardized instructions so that a salvage operation can be carried out promptly if a flap failure occurs. In this context, the use of artificial intelligence may help to simplify the interpretation of the values and increase their significance. In a previous study, a deep learning algorithm supported HSI was successfully tested [58], which could be transferred to the MFF monitoring in the near future.

## 5. Conclusions

As a valid, reproducible, examiner-independent, non-contact, and non-invasive measurement method, HSI provides a useful tool for perioperative MFF monitoring. In the event of MFF failure, the HSI shows early evidence of significant perfusion reduction, while the MDS signal, e.g., vascular blood flow through the anastomosis, still appears unchanged. A direct assessment of the anastomosis by HSI must be investigated in the future. Deploying HSI could further increase salvage rates and improve MFF success probability.

## Data Availability

All raw data on which this study is based will be made available by the corresponding author upon request.

## Supplementary Materials

The following supporting information can be downloaded at: https://www.mdpi.com/article/10.3390/jcm11144134/s1, Table S1: Mean values and standard deviation of StO2, NPI, THI, and TWI at time points t0 to t9, and comparison between the test (right) and control (left) sides. Superscripted asterisks indicate signif-icant differences. Table S2: Mean values and standard deviation of Max.KHz, Mean.KHz, Resistance Index (RI) and Pulsatility Index (PI) at time points t1 to t9, and comparison between the test (right) and control (left) sides. Superscripted asterisks indicate significant differences. Table S3: Difference between maximum and mean Doppler frequency.
