# Supplementary material for: Comparison of Hyperspectral Imaging and Microvascular Doppler for Perfusion Monitoring of Free Flaps in an In Vivo Rodent Model
Source: J Clin Med. 2022 Jul 16;11(14):4134. doi: 10.3390/jcm11144134 (PMC9321983; doi:10.3390/jcm11144134)
Supplement: Supplementary file 1 [file jcm-11-04134-s001.zip › jcm-1799780-supplementary.pdf]

Journal of Clinical Medicine

MDPI

Supplements

Table S1. Mean values and standard deviation of StO_2, NPI, THI, and TWI at time points t0 to t9, and comparison between the test (right) and control (left) sides. Superscripted asterisks indicate significant differences.

		StO_2			NPI			THI			TWI		
		Mean	±SD	P	Mean	±SD	P	Mean	±SD	P	Mean	±SD	P
**t0**	right	46.7	5.9	0.668	53.9	4.1	1.000	7.6	3.9	0.184	36.8	2.7	0.059
	left	47.1	5.8		53.9	3.6		8.9	3.4		38.6	3.7	
**t1**	right	60.5	5.8	0.077	62.4	3.1	0.004*	9.8	5.1	0.365	42.0	2.4	<0.001*
	left	56.7	8.4		58.9	4.5		10.8	3.9		39.8	1.9	
**t2**	right	27.9	4.4	0.419	41.8	3.5	0.183	9.2	6.2	0.854	39.7	3.6	0.465
	left	27.3	3.8		40.8	3.2		9.6	5.1		39.2	2.9	
**t3**	right	56.3	8.4	0.053	57.4	4.8	0.067	13.2	6.0	0.318	40.7	3.1	0.356
	left	50.1	12.8		53.8	7.9		14.5	5.3		40.1	2.3	
**t4**	right	54.4	6.8	0.451	56.7	3.3	0.258	11.9	8.0	0.651	41.7	2.7	0.920
	left	52.2	8.9		54.8	4.7		13.1	5.9		41.4	1.7	
**t5**	right	50.4	10.8	0.168	53.2	6.8	0.354	11.6	5.4	0.374	41.0	3.4	0.455
	left	50.1	8.9		53.4	5.5		10.7	5.7		41.0	2.0	
**t6**	right	51.8	10.2	0.053	53.7	6.3	0.132	10.5	5.7	0.259	40.7	3.9	0.901
	left	48.4	11.0		52.1	7.6		12.8	5.8		40.6	2.1	
**t7**	right	46.1	11.2	0.092	50.0	7.2	0.232	10.1	6.7	0.630	40.3	3.7	0.910
	left	45.0	10.7		49.5	8.0		12.0	5.3		40.0	2.0	
**t8**	right	46.1	10.9	0.118	49.7	7.1	0.415	12.1	6.5	0.343	40.5	3.7	0.343
	left	43.2	12.5		48.5	9.3		10.5	5.2		39.9	2.4	
**t9**	right	46.1	14.3	0.060	49.3	9.4	0.056	11.4	5.9	0.760	40.0	4.2	0.431
	left	41.8	14.4		46.9	10.6		10.7	4.7		39.1	1.7	

Table S2. Mean values and standard deviation of Max.KHz, Mean.KHz, Resistance Index (RI) and Pulsatility Index (PI) at time points t1 to t9, and comparison between the test (right) and control (left) sides. Superscripted asterisks indicate significant differences.

		**Max.KHz**			**Mean.KHz**			**RI**			**PI**		
		Mean	±SD	P	Mean	±SD	P	Mean	±SD	P	Mean	±SD	P
**t1**	right	7.3	0.5	0.463	4.7	1.0	0.421	0.6	0.1	0.286	1.1	0.5	0.586
	left	7.2	0.5		4.2	1.3		0.8	0.4		1.3	0.7	
**t2**	right	2.6	2.1	0.059	0.2	0.4	0.289	0.8	0.4	0.059	31.8	32.4	0.026*
	left	2.1	2.0		0.1	0.2		0.7	0.5		14.7	21.6	
**t3**	right	7.4	0.2	0.068	5.1	1.3	0.039*	0.6	0.2	0.069	1.1	0.7	0.052
	left	7.1	0.6		4.0	1.3		0.8	0.2		1.6	0.7	
**t4**	right	7.4	0.2	0.180	5.2	1.1	0.023*	0.6	0.2	0.009*	0.9	0.4	0.023*
	left	7.2	0.7		4.0	1.0		0.8	0.1		1.6	0.6	
**t5**	right	7.2	0.8	1.0	4.4	1.3	0.136	0.7	0.2	0.037*	1.2	0.5	0.084
	left	7.1	1.1		3.8	1.1		0.8	0.1		1.6	0.6	
**t6**	right	7.3	0.3	0.068	4.5	0.9	0.003*	0.7	0.1	0.005*	1.1	0.4	0.002*
	left	6.7	1.5		3.4	1.2		0.8	0.1		1.7	0.6	
**t7**	right	7.0	1.0	0.068	4.2	1.2	0.008*	0.7	0.1	0.026*	1.2	0.4	0.008*
	left	6.5	1.7		3.4	1.4		0.8	0.1		1.8	0.7	
**t8**	right	7.0	1.2	0.593	4.3	1.2	0.333	0.7	0.1	0.083*	1.2	0.4	0.202
	left	6.8	1.1		3.9	1.3		0.7	0.1		1.5	0.7	
**t9**	right	6.4	1.6	0.686	3.7	1.7	0.069	0.7	0.1	0.086	1.5	0.7	0.374
	left	6.1	1.6		3.3	1.4		0.8	0.1		1.7	0.8	

Table S3. Difference between maximum and mean Doppler frequency.

	Max.KHz right	Mean.KHz right	Diff.KHz (%)	P	Max.KHz left	Mean.KHz left	Diff.KHz (%)	P
t1	7.28	4.7256	2.5 (35.1)	<0.001	7.15	4.2	2.9 (41.2)	<0.001
t2	2.59	0.2367	2.3 (90.9)	<0.001	2.09	0.1	1.9 (95.2)	0.003
t3	7.38	5.0844	2.3 (31.1)	<0.001	7.08	4	3.1 (43.5)	<0.001
t4	7.39	5.192	2.2 (29.7)	<0.001	7.21	4	3.2 (44.5)	0.001
t5	7.16	4.4307	2.7 (38.1)	<0.001	7.08	3.8	3.3 (46.3)	0.002
t6	7.33	4.5167	2.8 (38.4)	0.002	6.68	3.4	3.3 (49.1)	0.002
t7	6.98	4.2358	2.7 (39.3)	0.002	6.53	3.4	3.1 (47.9)	0.003
t8	7.01	4.264	2.7 (39.2)	0.005	6.83	3.9	2.9 (42.9)	0.005
t9	6.36	3.7033	2.6 (41.8)	0.008	6.12	3.3	2.8 (46.1)	0.008
